# Supplementary material for: Disentangling the force of infection of SARS-CoV-2 in Dutch long-term care facilities
Source: BMC Public Health. 2025 Nov 10;25:3878. doi: 10.1186/s12889-025-25015-x (PMC12604230; doi:10.1186/s12889-025-25015-x)
Supplement: Supplementary file 1 — Supplementary Material 1. [file 12889_2025_25015_MOESM1_ESM.docx]

**Contributions to the force of infection of SARS-CoV-2 in Dutch long-term care facilities**

Mariken M. de Wit^1^, Marino van Zelst^#1^, Tjarda M. Boere^2^, Rolina D. van Gaalen^2^, Mart C. M. de Jong^1^, Albert Jan van Hoek^2^, Quirine ten Bosch^1^

^1^ Infectious Disease Epidemiology, Department of Animal Sciences, Wageningen University and Research

^2^ Center for Epidemiology and Surveillance of Infectious Diseases, National Institute for Public Health and the Environment (RIVM), Bilthoven, The Netherlands

^#^current address: Dutch Ministry of Health, Infectious Diseases Policy Department, Parnassusplein 5, 2511 VX Den Haag, the Netherlands

# Supplement

**S1 Text. Methods**

**S2 Text. Results: Model selection**

**S3 Text. Results: Model fit and diagnostics**

**S4 Text. Results: Resident-directed type-R estimates**

**S5 Text. Results: Absolute FOI estimates**

**S6 Text. Results: Results: Resident susceptibility estimates without variant adjustment**

**S7 Text. Results: Sensitivity analyses immunity assumptions**

**S8 Text. Results: Sensitivity analyses underreporting infections in general population**

**S9 Text. Results: Sensitivity analyses contact fractions**

## Methods

**Data preparation**

***Augmentation for LTCF-associated cases***

We augmented day of infection for resident and HCW cases to be able to calculate LTCF-level prevalence of infectious individuals. The day of infection was estimated based on available information on the timing of symptom onset or diagnostic test result.

*Day of infection.*

Following the observation process based of the LTCF reactive testing policy, for the first reported case in an outbreak, we augmented their day of infection based on the day of symptom onset. We reconstructed the date of infection by subtracting the incubation period from the day of symptom onset. We assumed the incubation period to be log-normally distributed with location parameter μ = 1.6, and scale parameter σ = 0.5 (median: 5 days) [1]. The duration of the incubation period was obtained by a random draw from this distribution. The day of infection was then set as the day of symptom onset minus the incubation period

As the detection of a case led to the testing of all other residents, we used the day of positive test result for all subsequent people in same outbreak and for those with unknown day of symptoms onset. From the date of positive test result we reconstructed the date of infection using a normalized version of the probabilistic distribution of PCR positive test probability over time constructed by Hellewell et al. [2] (see *Supplementary Figure 1*). This distribution assumes that all exposures are detected between 0 and 30 days since exposure. Again, the duration of this period was obtained by a random draw from this distribution. The day of infection was het set as the day of positive test result minus this delay period.

*Start and duration of infectiousness*.

We assumed that all cases started their infectious period three days after their day of infection [3] and that it lasted for five days, after which people start isolating. The duration of infectiousness was based on the difference between the latent period and incubation period (see above, 2 day difference), and time between symptom onset and positive test notification in the Netherlands (3 days) [4].

*LTCF identifier.*

The LTCF identifier provides information about which LTCF someone lives or works. It was only available for a subset of residents (68%) and missing for HCWs. We imputed the LTCF identifier when missing for residents and HCWs by matching their cluster identifier to those from residents with recorded LTCF identifier. Cluster identifiers indicate which cases were linked together in space and time. Individuals across different LTCFs could be part of the same transmission cluster. When multiple LTCFs were associated with one cluster, we randomly selected, with equal weights, one of the LTCFs that were part of the cluster. This random selection was performed for each (augmented) dataset and thereby contributed to the variation between datasets.

***General population***

Missing information for the general population was imputed rather than augmented, because the number of cases in the general population was much higher than for LTCF-related cases thereby reducing the impact of individual variability on the outcomes. For those with recorded day of symptom onset, the date of infection was assumed to be five days earlier. This corresponds to the mean of the incubation period distribution described above. For those with recorded day of positive test result, the date of infection was assumed to be seven days earlier. This was based on the mean interval between symptom onset and laboratory test result in the Netherlands (2 days) [4]. The start and duration of infectiousness was assumed equal to that of the LTCF-associated cases. We assumed an underreporting of cases in the general population of about 50%. This was estimated by comparing incidence estimates based on hospitalization data to the number of positive tests [5, 6]. Therefore, reported case numbers were multiplied by two to account for asymptomatic and unreported infections. We assessed the impact of this assumption in a sensitivity analysis.

We also required information on population sizes of each group to obtain daily prevalence estimates. Given the 1 to 0.98 resident to HCW ratio in Dutch LTCFs [7], we set the population size of residents and that of HCW equal to the LTCF capacity. To ensure the estimated prevalence in the general population better reflects the prevalence in those coming into contact with LTCF residents, we weighted age-specific prevalence estimates by the proportion of resident contacts within each age-group (see *Supplementary Table 1*).


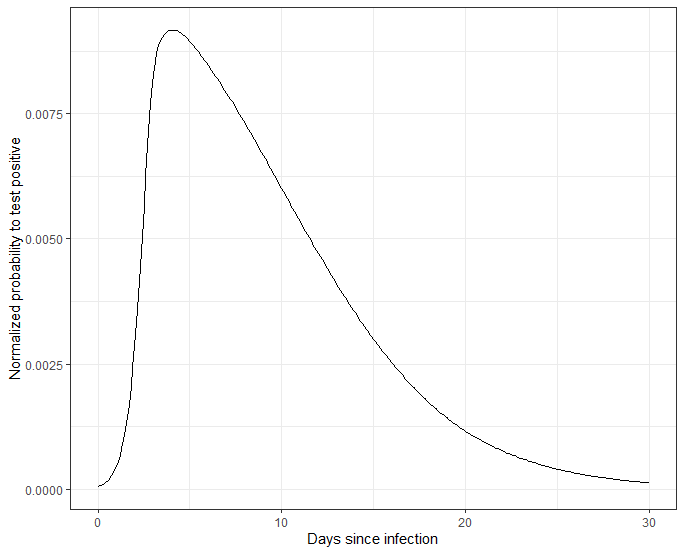


*Supplementary Figure 1: Distribution of time between infection and positive PCR test, adapted from Hellewell et al 2021.*

**Age distribution of resident contacts**

We analyzed data published in a contact study of people aged 70+ [8]. A total of 271 contacts were reported. The full age distribution is presented in Supplementary Figure *2* with the associated table for the age-distribution as used in the data from the general population in Supplementary Table 1.


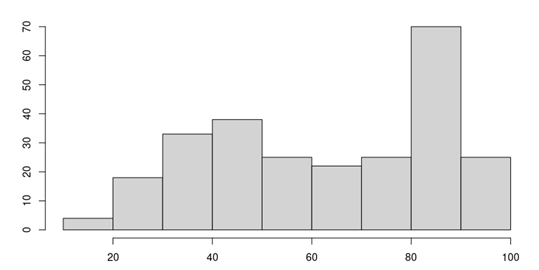


*Supplementary Figure 2: Age distribution of contacts of people aged 70+*

| **Age in years** | **Proportion of resident contacts** |
| --- | --- |
| **0-19** | 0.011 |
| **20-29** | 0.050 |
| **30-29** | 0.112 |
| **40-79** | 0.435 |
| **80+** | 0.392 |

*Supplementary Table 1: Contact matrix of LTCF resident contacts*

**Estimation of time-varying transmission rate parameters**

We considered that group-specific transmission rate parameters may have differed over time:

$\beta_{i}\left( t \right)={exp}^{c_{0}+c_{i}x_{1}+c_{2}x_{2}\ldots c_{n}x_{n}}.$ (Equation S1)

Here, $x_{n}$ and *c_n_* denote respectively the dummy variables and coefficients corresponding to each period *t*, with group 0 being the reference group whose effect is absorbed in the intercept c_0_. Equation S1 implies that temporal changes in $\beta_{i}(t)$ were similar between groups. To relax this assumption, interaction terms between period and groups can be included.

**Estimating relative susceptibility and infectivity**

Relative susceptibility of residents and infectivity of HCW and the general population was estimated from the GLM coefficients. The susceptibility of residents (relative to period A) was obtained for each period by taking the exponent of the period coefficient ($c_{p}$). The infectivity of HCW was obtained by taking the exponent of the HCW coefficient ($c_{HCW}$). The same logic was applied to the general population, where the exponent of the general population coefficient ($c_{gen pop}$) was added to the exponent of the interaction term (${c_{gen pop\_period\_c}, c}_{gen pop_{period}\_d})$where applicable.

Adjustments for increased infectiousness of Alpha and Delta variants were made by dividing the resident susceptibility values by the increase in infectiousness (i.e., 1.7 in period B and C, 2 in period D and E).

## Results: Model selection

We compared WAIC values between models assuming different distributions in the data. Both the beta-binomial and the beta binomial zero-inflated distribution showed a large improvement compared to the binomial and binomial zero-inflated distribution, see Supplementary Figure 3. As WAIC estimates using the beta-binomial and beta-binomial zero-inflated distributions were similar, the simplest model (beta-binomial distribution) was chosen.


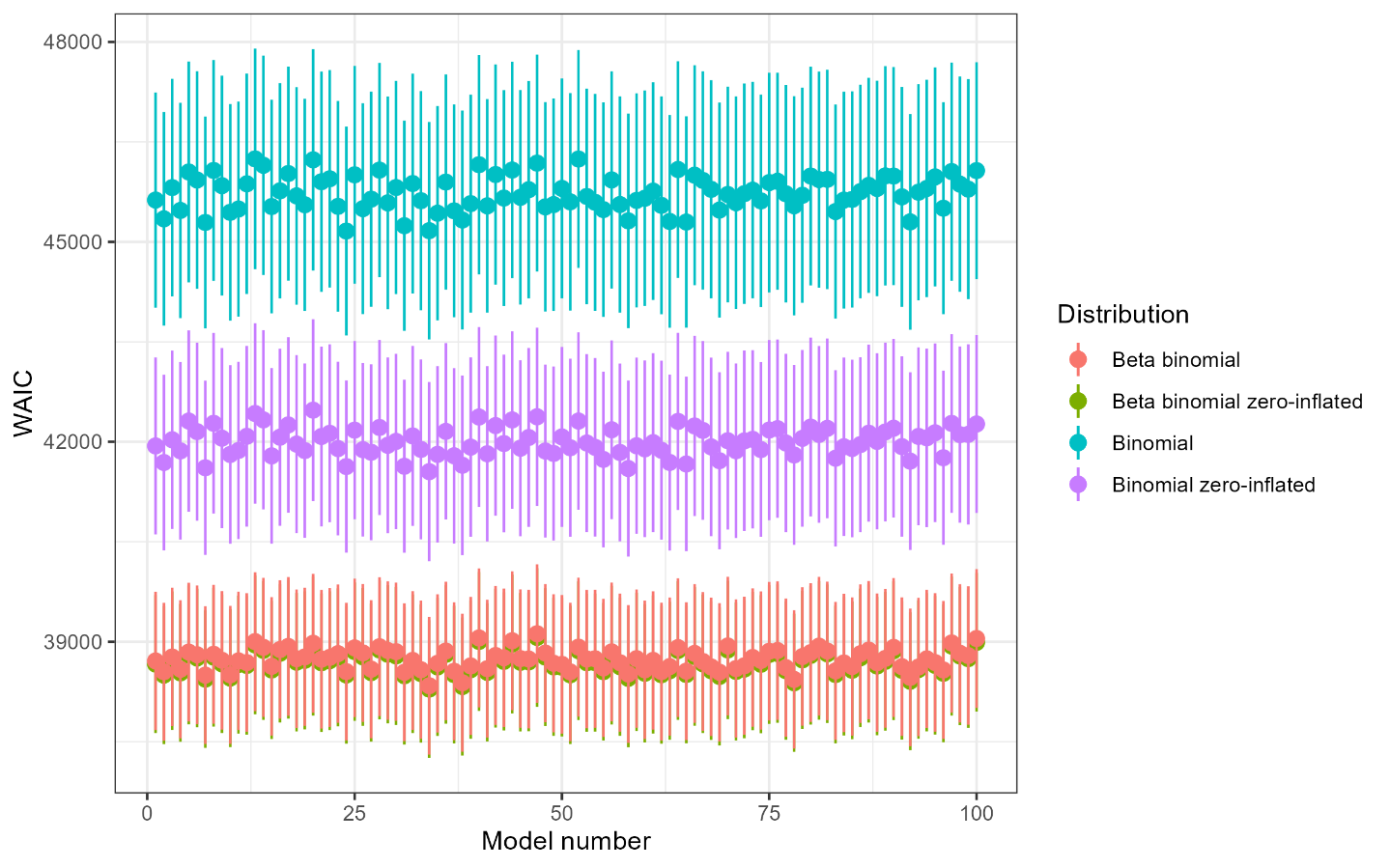


*Supplementary Figure 3: WAIC for each fitted model using different distributions. Vertical lines represent 95% confidence intervals*

Next, we compared WAIC values to assess the inclusion of period as an explanatory variable and its interaction with each group (i.e., HCW and general population). Models including a period term performed better than the null model, see Supplementary Figure 4. WAIC estimates of models with interaction terms performed better than those without, with the lowest WAIC values observed for the model only including the significant interaction terms. These were period C with the general population and period D with the general population. All further analyses were conducted on the model using the beta-binomial distribution including period as explanatory variable and these two interaction terms.


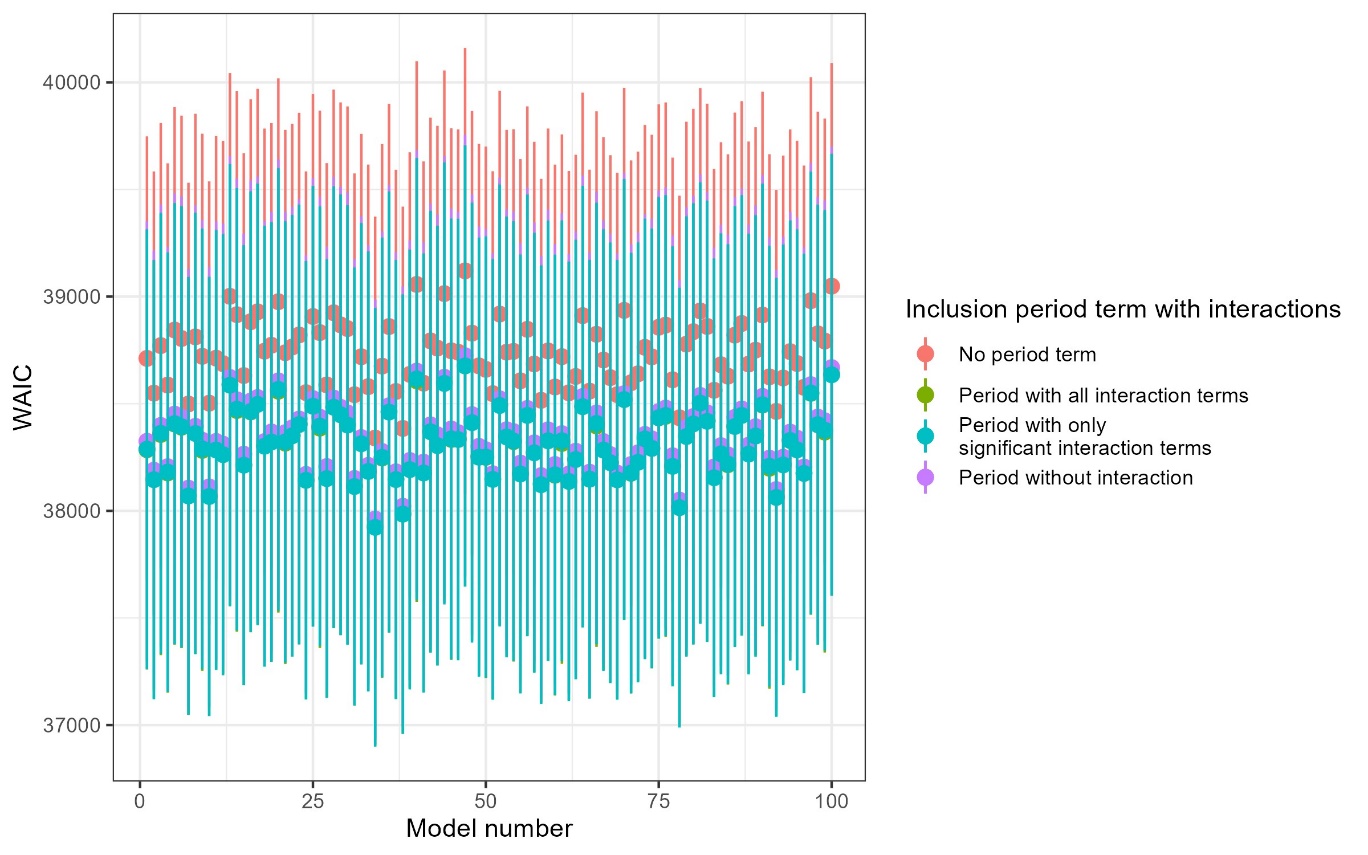


Supplementary Figure 4: WAIC for each fitted model using different model formulations for the inclusion of period as explanatory variable with its interactions. Vertical lines represent 95% confidence intervals.

## Results: Model fit and diagnostics

In the main text model fit was assessed by comparing daily observed and predicted number of infections (main text figure 2A), showing the 7-day average. A version showing daily results, without taking a multi-day average, is shown in Supplementary Figure 5.


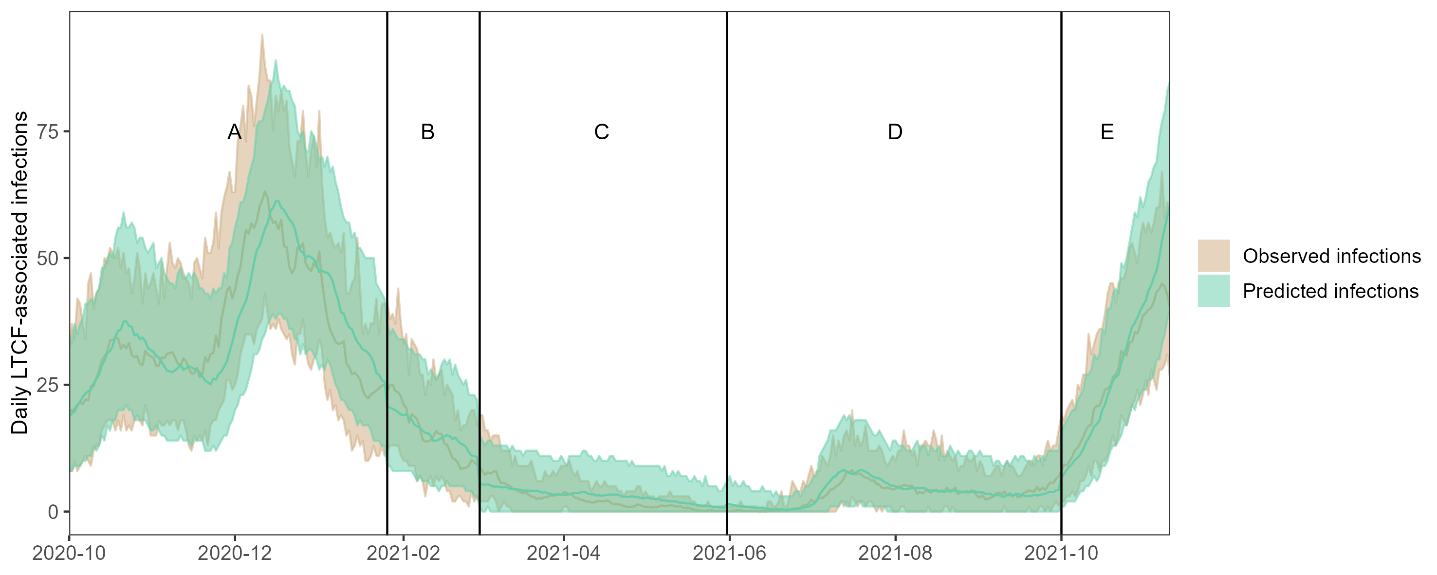


Supplementary Figure 5: Comparison between the daily number of observed infections (in the data) and predicted infections (from the model)**.**. Dark lines indicate mean values. Shaded areas show 95% prediction intervals obtained by generating 1000 predictions by randomly sampling from the posterior distributions and augmented datasets.

In addition to assessing model fit by comparing daily observed and predicted number of infections, we also compared the total observed and predicted number of infections per LTCF during the study period, see Supplementary Figure 6. Observations and predictions showed good agreement or small and medium number of infections, but sizes of the largest outbreaks were underestimated.


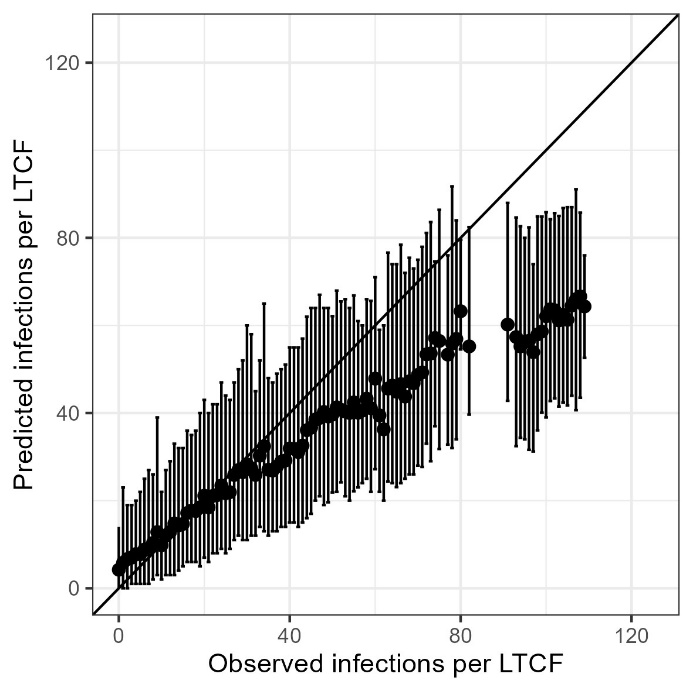


Supplementary Figure 6: Comparison between number of observed infections per LTCF (in the data) and predicted infections per LTCF (from the model) over the full study period. The diagonal line indicates the points for which the observed value is equal to the predicted value. Dots indicate the mean predicted value. Errors bars represent 95% prediction intervals obtained by generating 1000 predictions by randomly sampling from the posterior distributions and augmented datasets.

Traceplots, correlation plots and posterior density plots are shown below.


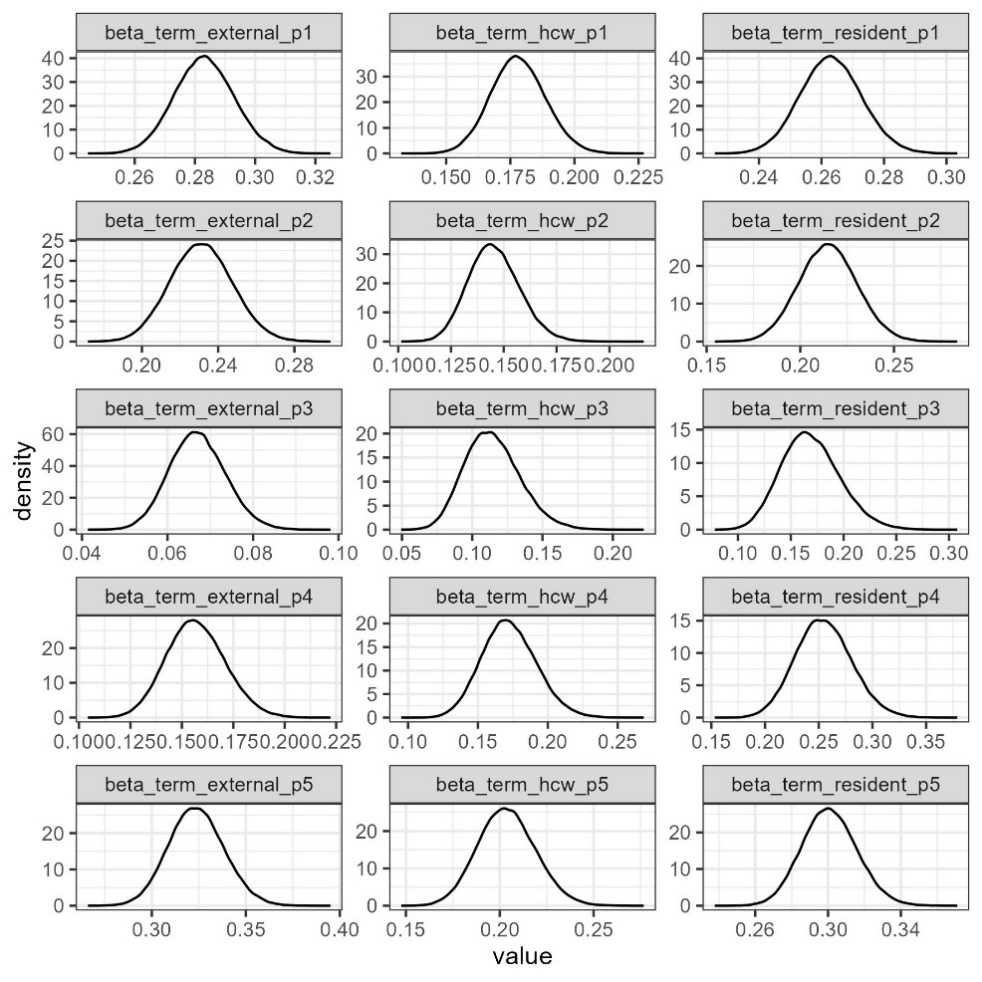


Supplementary Figure 7: Posterior density plots of all transmission rate parameters


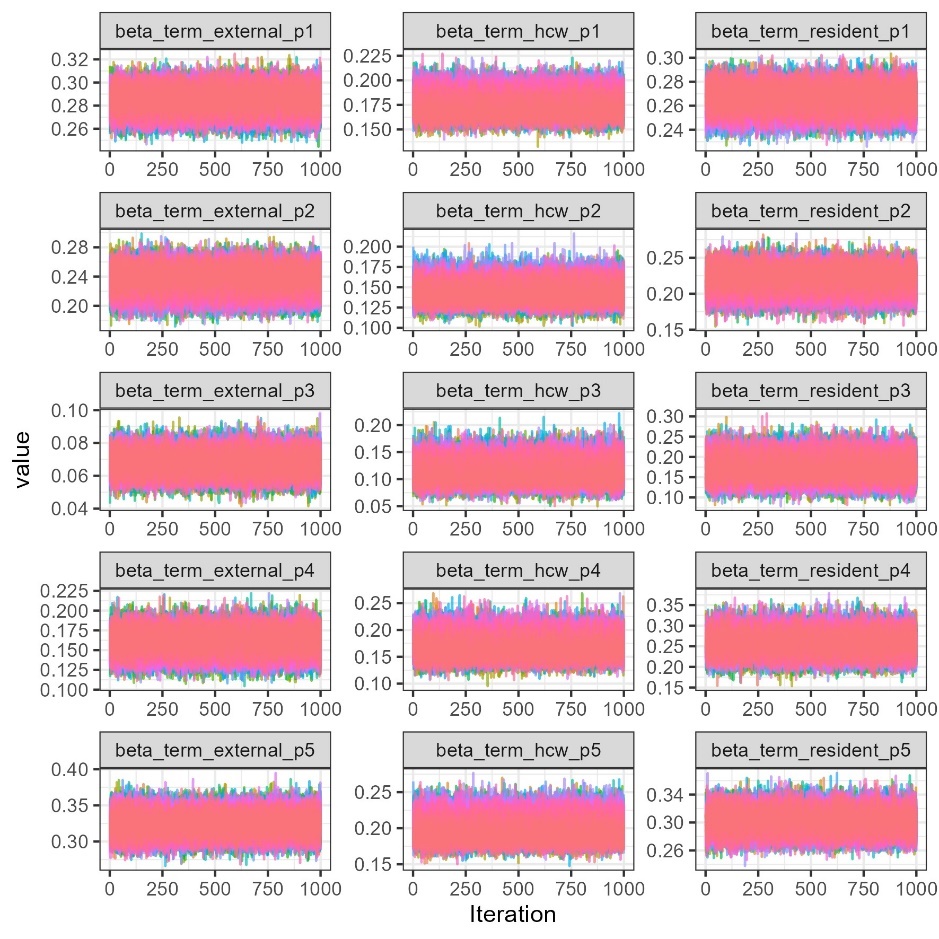


Supplementary Figure 8: Traceplots of all transmission rate parameters


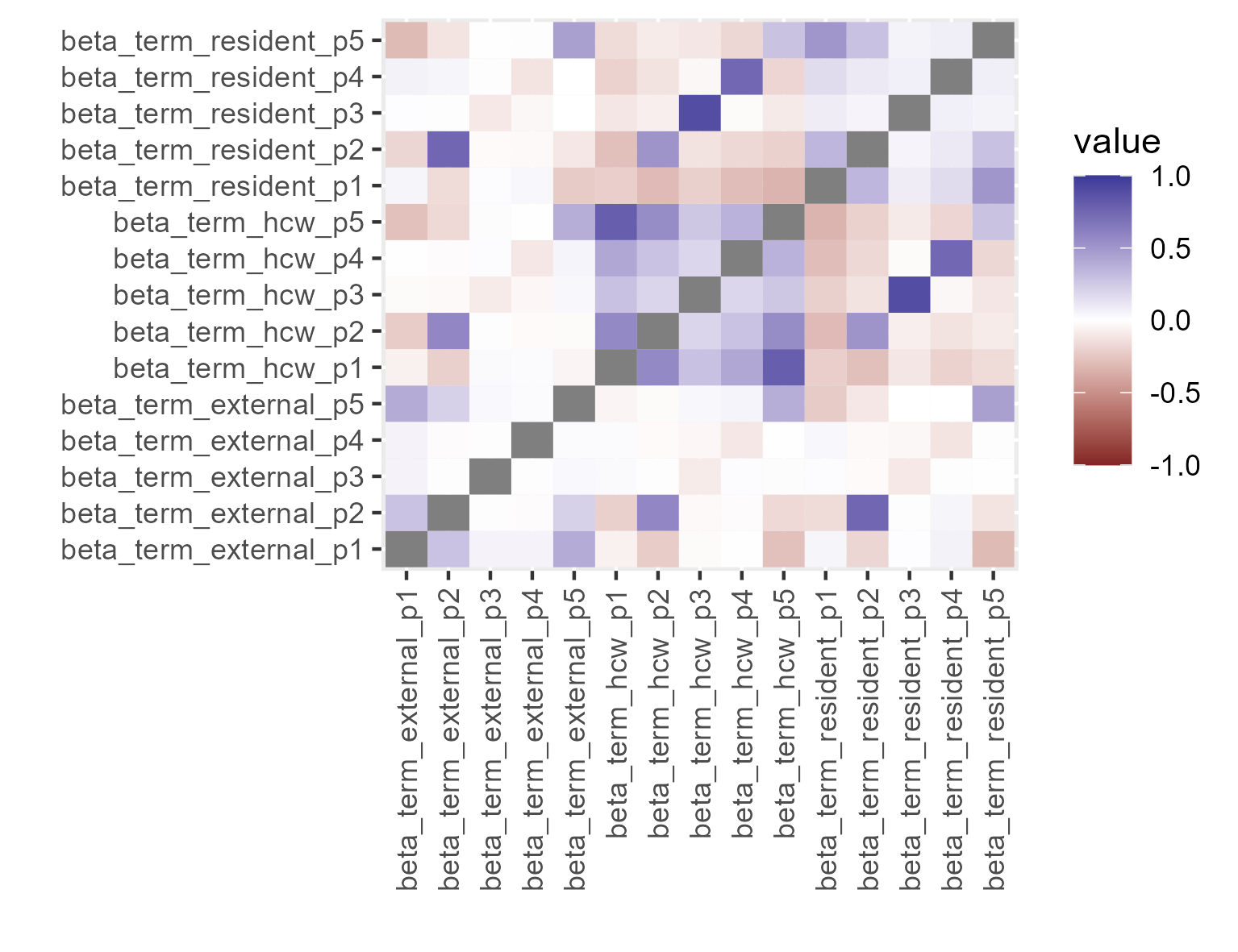


Supplementary Figure 9: Correlation plot of transmission rate parameters

## Results: Group-specific transmission rate parameter estimates

The posterior values of the group-specific transmission rate parameters $\beta_{i}(t)$ multiplied by the contact fraction ${Fr}_{i}$are presented below for both the base model (not including variation over time) and the final (period) model.

Supplementary Table 2: Estimates of group-specific transmission rate parameters multiplied by the contact fraction ($\beta_{i}\left( t \right)*{Fr}_{i}$) for the base model and the main period model, both using the beta binomial distribution. Mean WAIC is calculated as the mean across all augmented datasets (n=100). Phi is the overdispersion parameter.

|  | **Base model** | **Period model** | | | | |
| --- | --- | --- | --- | --- | --- | --- |
|  |  | **Period A** | **Period B** | **Period C** | **Period D** | **Period E** |
| **Resident** | 0.09  (0.08-0.10) | 0.09  (0.08-0.09) | 0.07  (0.06-0.08) | 0.06  (0.04-0.08) | 0.08  (0.07-0.10) | 0.10  (0.09-0.11) |
| **HCW** | 0.06  (0.05-0.07) | 0.06  (0.05-0.07) | 0.05  (0.04-0.06) | 0.04  (0.03-0.05) | 0.06  (0.04-0.07) | 0.07  (0.06-0.08) |
| **General population** | 0.07  (0.07-0.08) | 0.09  (0.09-0.10) | 0.08  (0.07-0.09) | 0.02  (0.02-0.03) | 0.05  (0.04-0.06) | 0.11  (0.10-0.12) |
| **Mean WAIC** | 38,733 | 38,314 | | | | |
| **Phi** | 88  (82-96) | 89  (82-96) | | | | |

## Results: Absolute FOI estimates

In addition to the relative FOI estimates shown in main text figure 2C, the absolute values are shown below.


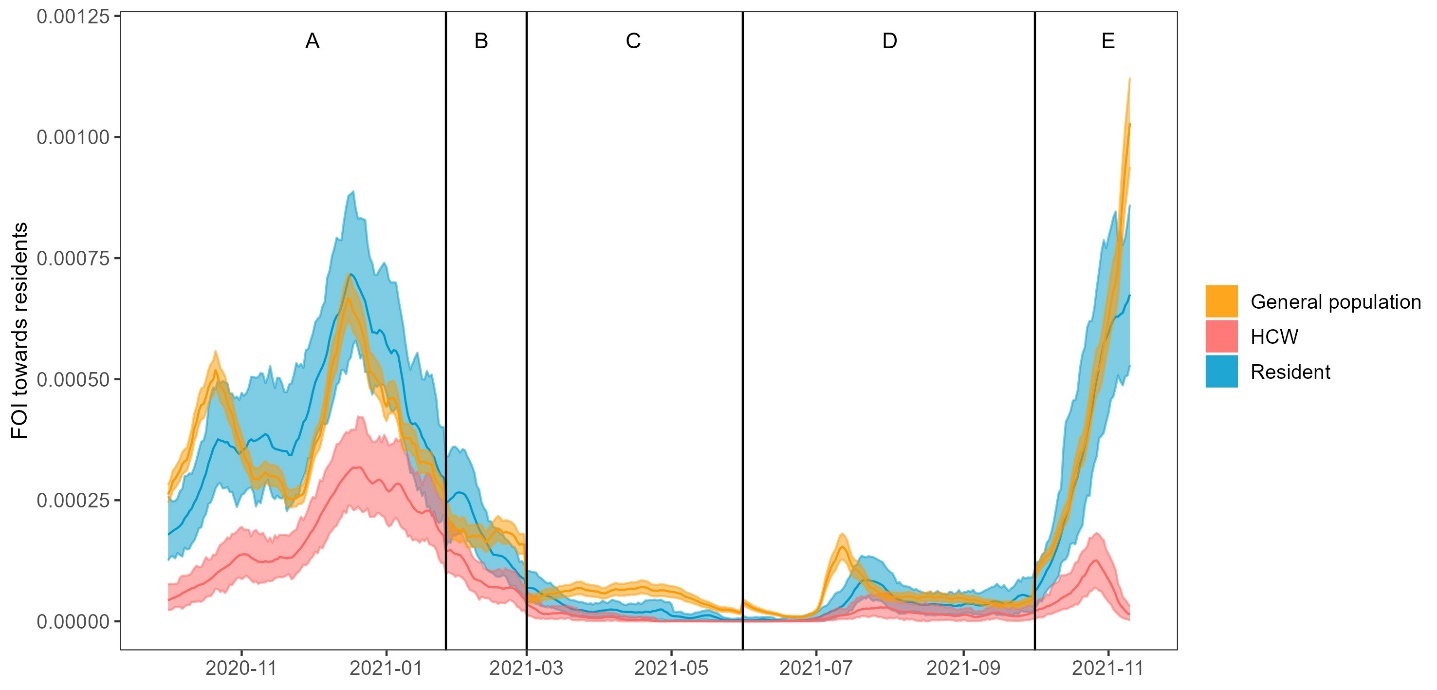


*Supplementary Figure 10: Daily force of infection from each LTCF group towards residents. Dark lines indicate mean values. Shaded areas show 95% prediction intervals obtained by generating 1000 predictions by randomly sampling from the posterior distributions and datasets.*

## Results: Resident susceptibility estimates without variant adjustment

Estimates for the resident susceptibility compared presented in the main text were adjusted for increased transmission success of newly emerging variants. Unadjusted susceptibility estimates are shown in Supplementary Figure 11.


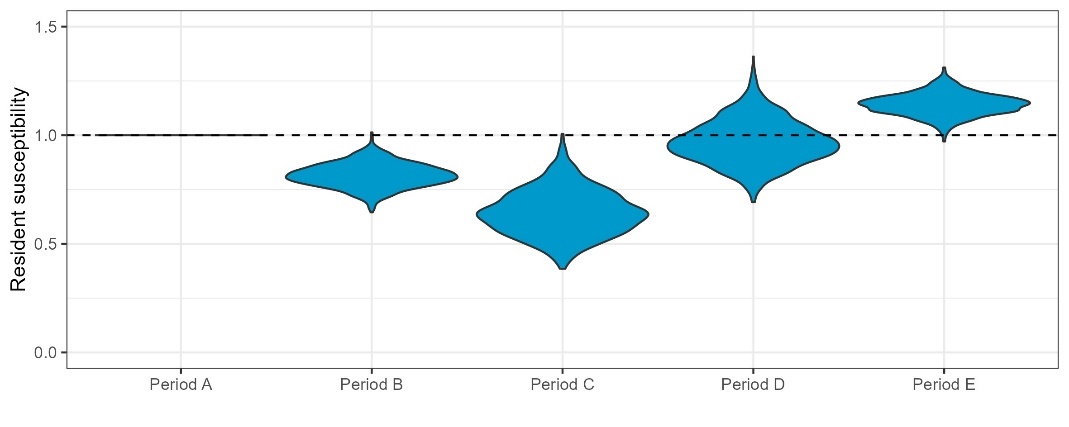


*Supplementary Figure 11: Relative susceptibility to infection in residents, unadjusted for increased transmissibility from newly emerging variants. The horizontal line at y=1 indicates no difference compared to period A.*

## Results: Sensitivity analyses immunity assumptions

We examined the impact of assumptions made about the size of the susceptible resident population. This is determined by model assumptions on the build-up of immunity in LTCF residents (i.e., whether complete immunity after infection is assumed), and the immune status of newly admitted residents (i.e., theoretically, following death of residents). The base scenario, as used in the main text, assumed imperfect immunity after infection, and that newly admitted residents were on average as susceptible as the resident population. In the sensitivity analysis, we modelled an extreme scenario where immunity after infection was perfect and lasting, and newly admitted residents were also immune. We assumed that (theoretical) new admissions happened on the same day following the death of residents.

This scenario differs in the number of susceptible people in each LTCF over time. For the baseline scenario, assuming imperfect immunity, the mean number of susceptible residents at an LTCF was 84, the median was 71. When assuming permanent, perfect immunity after infection with new residents also being immune, the mean was 75 and the median 62.

*Supplementary Table 3:* *Estimates of group-specific transmission rate parameters multiplied by the contact fraction (*$\beta_{i}\left( t \right)*{Fr}_{i}$*) for the period model, using the beta binomial distribution. Immunity is assumed to be perfect and lasting, and new admissions comprise recovered residents. Phi is the overdispersion parameter.*

|  | **Period model** | | | | |
| --- | --- | --- | --- | --- | --- |
|  | **Period A** | **Period B** | **Period C** | **Period D** | **Period E** |
| **Resident** | 0.10  (0.10-0.11) | 0.10  (0.08-0.11) | 0.07  (0.05-0.10) | 0.10  (0.8-0.13) | 0.13  (0.12-0.14) |
| **HCW** | 0.07  (0.06-0.08) | 0.07  (0.06-0.08) | 0.05  (0.03-0.07) | 0.07  (0.06-0.09) | 0.09  (0.08-0.10) |
| **General population** | 0.10  (0.10-0.11) | 0.10  (0.08-0.11) | 0.03  (0.02-0.03) | 0.06  (0.05-0.08) | 0.13  (0.12-0.14) |
| **Phi** | 68  (63-74) | | | | |


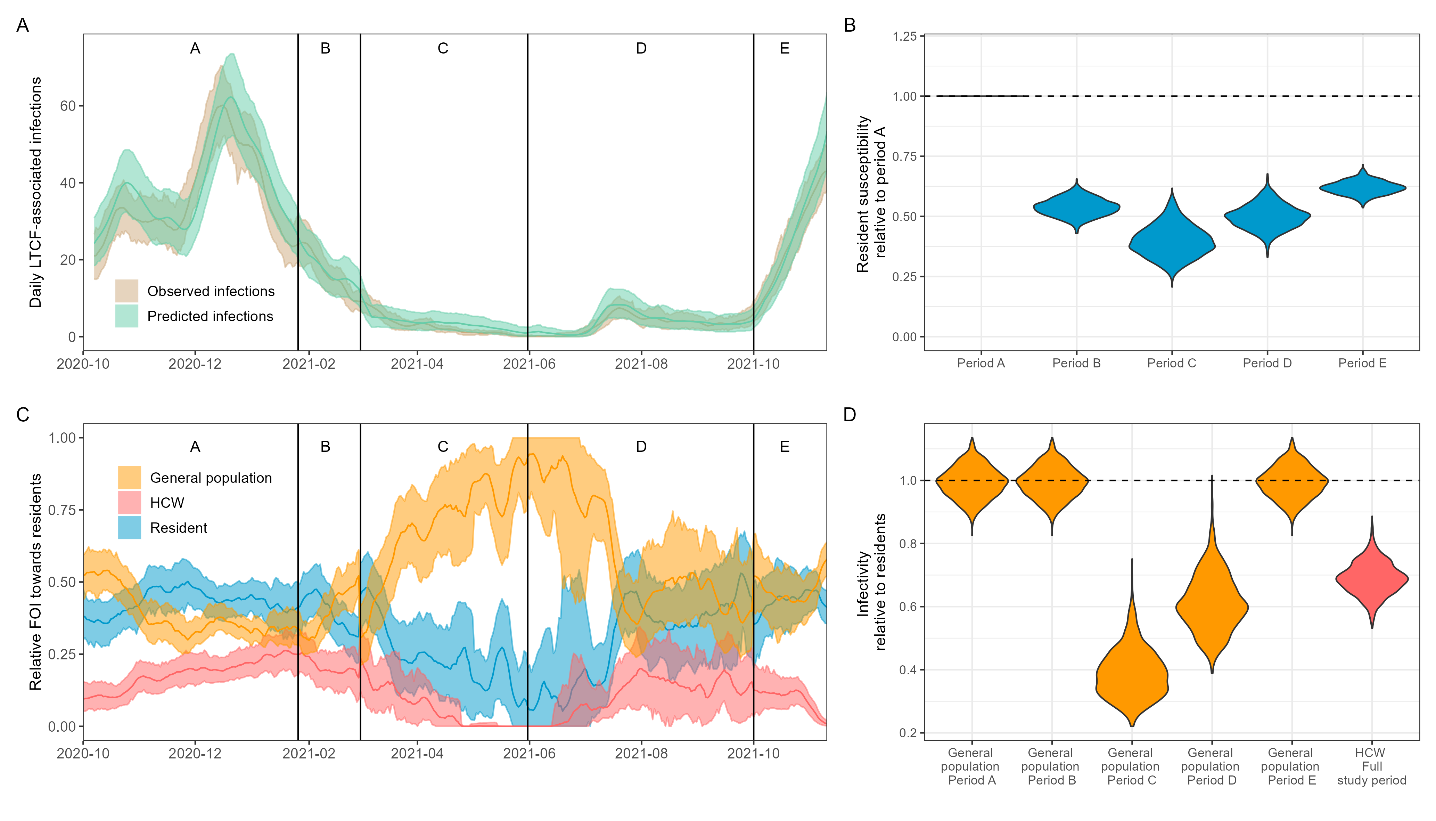


Supplementary Figure 12: Model fits and outcomes for the alternative model assuming permanent and perfect immunity (sensitivity analysis).

**A) Comparison between the daily number of observed infections (in the data) and predicted infections (from the model).** Values of the 7-day average are shown. Dark lines indicate mean values. Shaded areas show 95% prediction intervals obtained by generating 1000 predictions by randomly sampling from the posterior distributions and augmented datasets.

**B) Relative susceptibility to infection in residents.** The horizontal line at y=1 indicates no difference compared to period A.

**C) Daily relative force of infection from each LTCF group towards residents**. Dark lines indicate mean values. Shaded areas show 95% prediction intervals obtained by generating 1000 predictions by randomly sampling from the posterior distributions and datasets.

**D) Relative infectivity compared to residents.** The horizontal line at y=1 indicates an infectivity equal to that of residents. Estimates for the infectivity of the general population differed in periods C and D compared to all other periods as these were significant interaction terms in the statistical model.


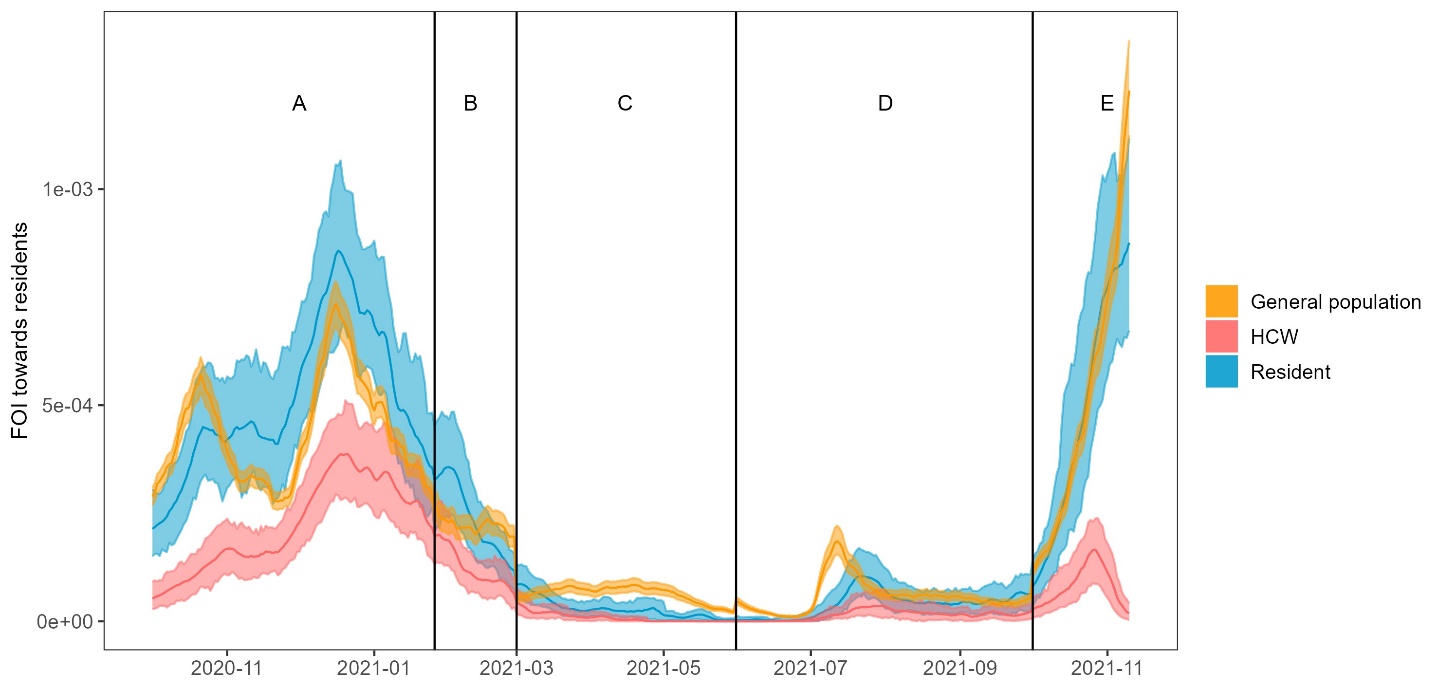


Supplementary Figure 13: Daily force of infection from each LTCF group towards residents for the alternative model assuming permanent and perfect immunity (sensitivity analysis). Dark lines indicate mean values. Shaded areas show 95% prediction intervals obtained by generating 1000 predictions by randomly sampling from the posterior distributions and datasets.


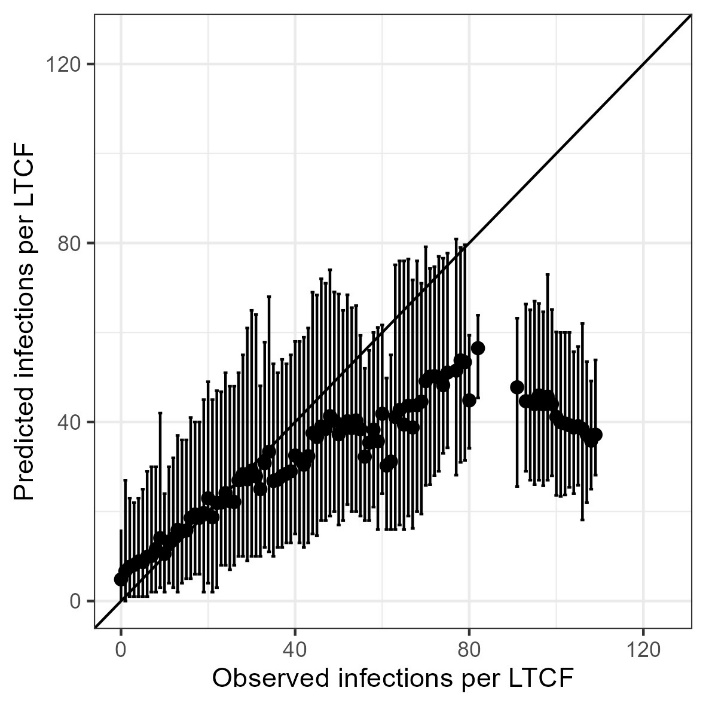


Supplementary Figure 14: Comparison between number of observed infections per LTCF (in the data) and predicted infections per LTCF (from the model) over the full study period, for the alternative model assuming permanent and perfect immunity (sensitivity analysis). The diagonal line indicates the points for which the observed value is equal to the predicted value. Dots indicate the mean predicted value. Errors bars represent 95% prediction intervals obtained by generating 1000 predictions by randomly sampling from the posterior distributions and augmented datasets.

## Results: Sensitivity analyses underreporting infections in general population

We examined the impact of assumptions made about the extent of underreporting of infections in the general population. In the main text, we assumed that 50% of infections went unreported and therefore multiplied the number of infections by 2. Here, we assumed that 75% of infections went undetected and we therefore multiplied the number of infections by 4.

Results showed that while estimates for the group-specific transmission rate parameters remain mostly unchanged for residents and HCW, the values have reduced by around 50% for the general population. The same reduction was observed in the estimated infectivity of the general population compared to the residents. All other outcome measures, including the contributions from the general population to the FOI experienced by residents were not affected by variation in underreporting rate.

*Supplementary Table 4: Estimates of group-specific transmission rate parameters multiplied by the contact fraction (*$\beta_{i}\left( t \right)*{Fr}_{i}$*) for the period model, using the beta binomial distribution, assuming a 75% underreporting rate of infections in the general population. Phi is the overdispersion parameter.*

|  | **Period model** | | | | |
| --- | --- | --- | --- | --- | --- |
|  | **Period A** | **Period B** | **Period C** | **Period D** | **Period E** |
| **Resident** | 0.09  (0.08-0.10) | 0.07  (0.06-0.08) | 0.06  (0.04-0.09) | 0.09  (0.07-0.11) | 0.10  (0.09-0.11) |
| **HCW** | 0.06  (0.05-0.06) | 0.05  (0.04-0.05) | 0.04  (0.03-0.06) | 0.06  (0.04-0.07) | 0.06  (0.05-0.08) |
| **General population** | 0.05  (0.04-0.05) | 0.04  (0.03-0.05) | 0.01  (0.01-0.01) | 0.03  (0.02-0.03) | 0.05  (0.05-0.06) |
| **Phi** | 89  (82-96) | | | | |


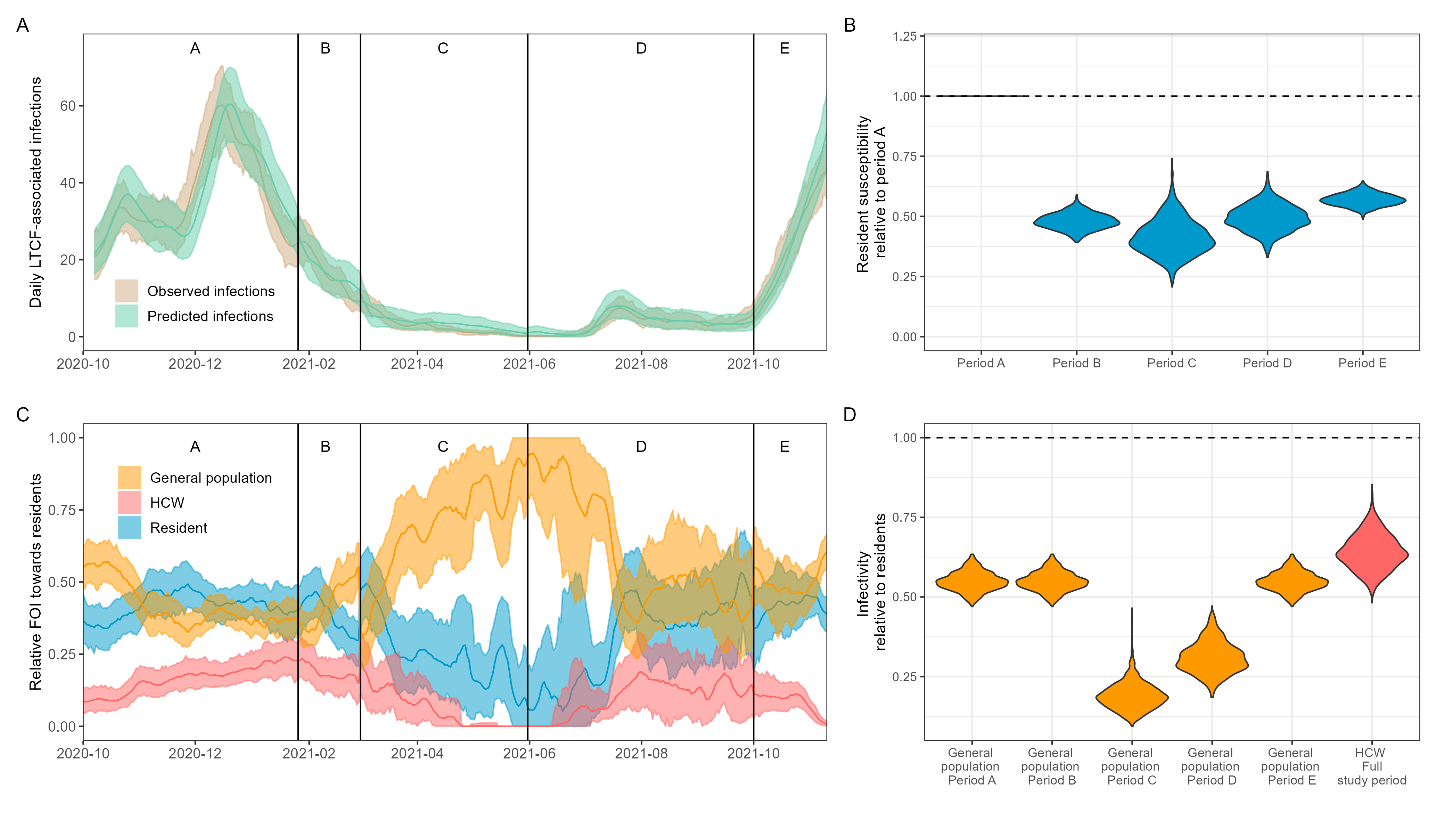


Supplementary Figure 15: Model fits and outcomes for the alternative model assuming a 75% underreporting rate of infections in the general population.

**A) Comparison between the daily number of observed infections (in the data) and predicted infections (from the model).** Values of the 7-day average are shown. Dark lines indicate mean values. Shaded areas show 95% prediction intervals obtained by generating 1000 predictions by randomly sampling from the posterior distributions and augmented datasets.

**B) Relative susceptibility to infection in residents.** The horizontal line at y=1 indicates no difference compared to period A.

**C) Daily relative force of infection from each LTCF group towards residents**. Dark lines indicate mean values. Shaded areas show 95% prediction intervals obtained by generating 1000 predictions by randomly sampling from the posterior distributions and datasets.

**D) Relative infectivity compared to residents.** The horizontal line at y=1 indicates an infectivity equal to that of residents. Estimates for the infectivity of the general population differed in periods C and D compared to all other periods as these were significant interaction terms in the statistical model.


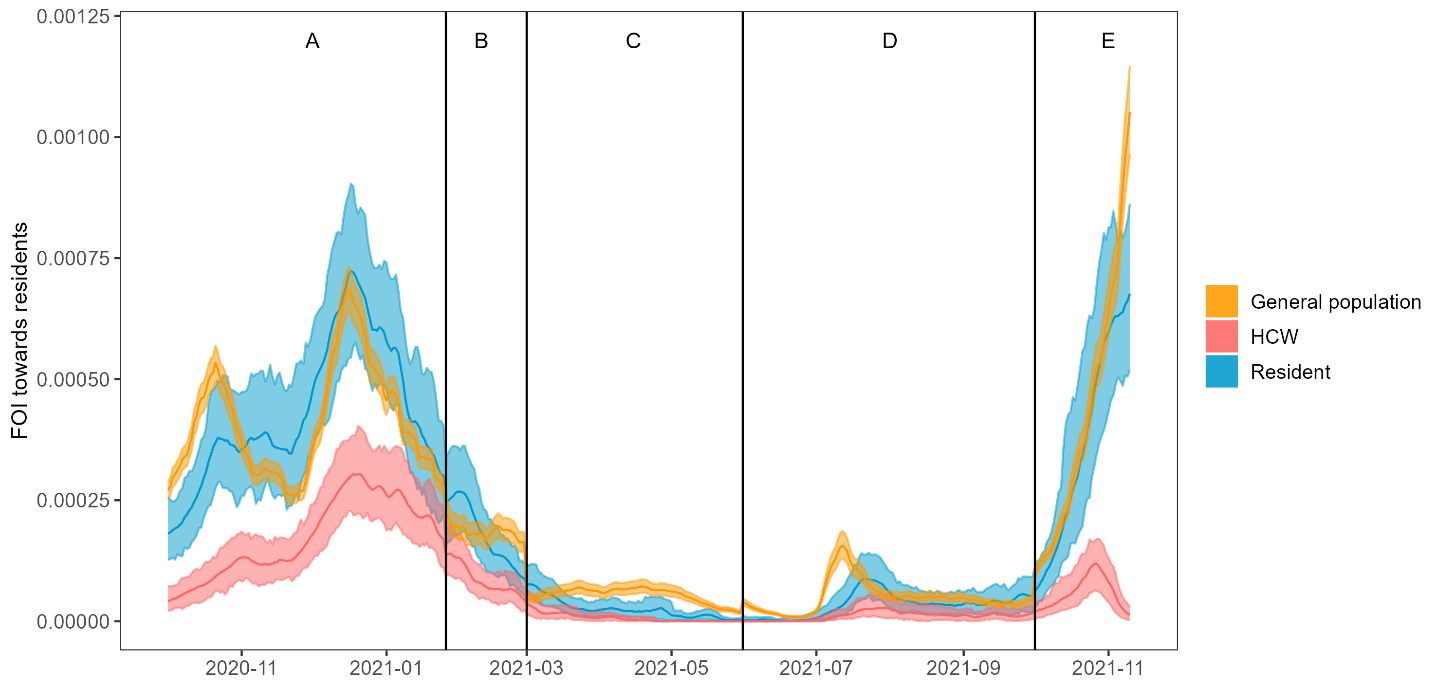


Supplementary Figure 16: Daily force of infection from each LTCF group towards residents for the alternative model assuming a 75% underreporting rate of infections in the general population. Dark lines indicate mean values. Shaded areas show 95% prediction intervals obtained by generating 1000 predictions by randomly sampling from the posterior distributions and datasets.

## Results: Sensitivity analyses contact fractions

We examined the impact of assumptions made about the distribution of resident contacts across the three groups. In the main text, contacts were equally distribution (1/3 each). Here, we assumed that 30% of resident contacts are with other residents, 60% of resident contacts are with HCW, and 10% of resident contacts are with the general population.

Results showed that estimates for the resident-directed effective reproduction number and relative as well as absolute FOI remain unchanged. The relative infectivity of the general population and HCW compared to residents is however impacted by variation in contact fraction. A reduction in contacts led to a higher relative infectivity and vice versa.

*Supplementary Table 5: Estimates of group-specific transmission rate parameters multiplied by the contact fraction (*$\beta_{i}\left( t \right)*{Fr}_{i}$*) for the period model, using the beta binomial distribution, assuming unequal resident contact fractions (30% residents, 60% HCW, 10% general populations). Phi is the overdispersion parameter.*

|  | **Period model** | | | | |
| --- | --- | --- | --- | --- | --- |
|  | **Period A** | **Period B** | **Period C** | **Period D** | **Period E** |
| **Resident** | 0.09  (0.09-0.10) | 0.08  (0.07-0.09) | 0.06  (0.04-0.07) | 0.09  (0.07-0.10) | 0.11  (0.10-0.12) |
| **HCW** | 0.07  (0.07-0.08) | 0.06  (0.05-0.07) | 0.04  (0.03-0.06) | 0.06  (0.05-0.08) | 0.08  (0.07-0.09) |
| **General population** | 0.09  (0.09-0.10) | 0.07  (0.06-0.08) | 0.02  (0.02-0.03) | 0.05  (0.04-0.06) | 0.10  (0.10-0.11) |
| **Phi** | 90  (83-98) | | | | |


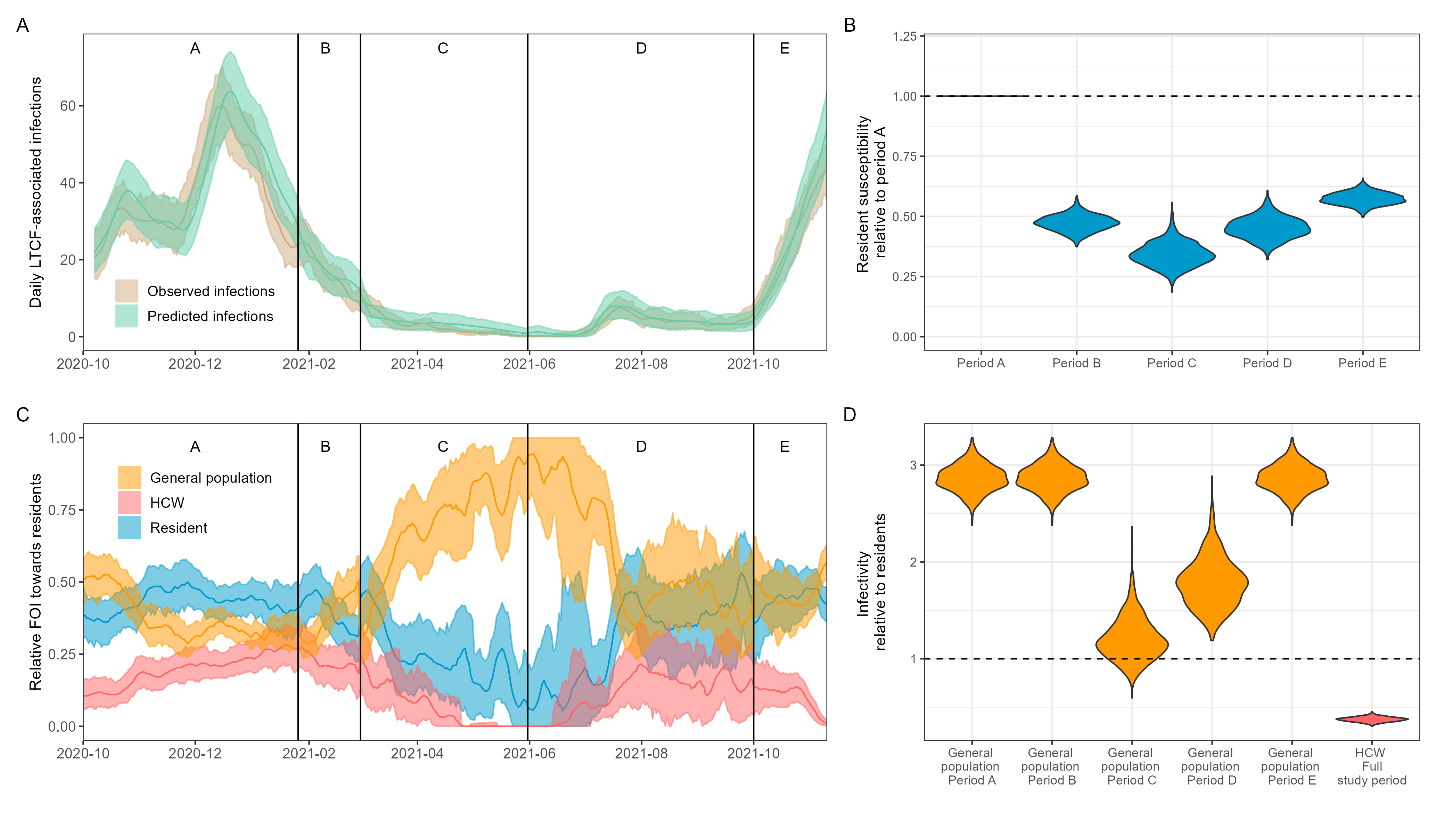


Supplementary Figure 17: Model fits and outcomes for the alternative model assuming unequal resident contact fractions (30% residents, 60% HCW, 10% general populations).

***A) Comparison between the daily number of observed infections (in the data) and predicted infections (from the model).*** *Values of the 7-day average are shown. Dark lines indicate mean values. Shaded areas show 95% prediction intervals obtained by generating 1000 predictions by randomly sampling from the posterior distributions and augmented datasets.*

**B) Relative susceptibility to infection in residents.** The horizontal line at y=1 indicates no difference compared to period A.

**C) Daily relative force of infection from each LTCF group towards residents**. Dark lines indicate mean values. Shaded areas show 95% prediction intervals obtained by generating 1000 predictions by randomly sampling from the posterior distributions and datasets.

**D) Relative infectivity compared to residents.** The horizontal line at y=1 indicates an infectivity equal to that of residents. Estimates for the infectivity of the general population differed in periods C and D compared to all other periods as these were significant interaction terms in the statistical model.


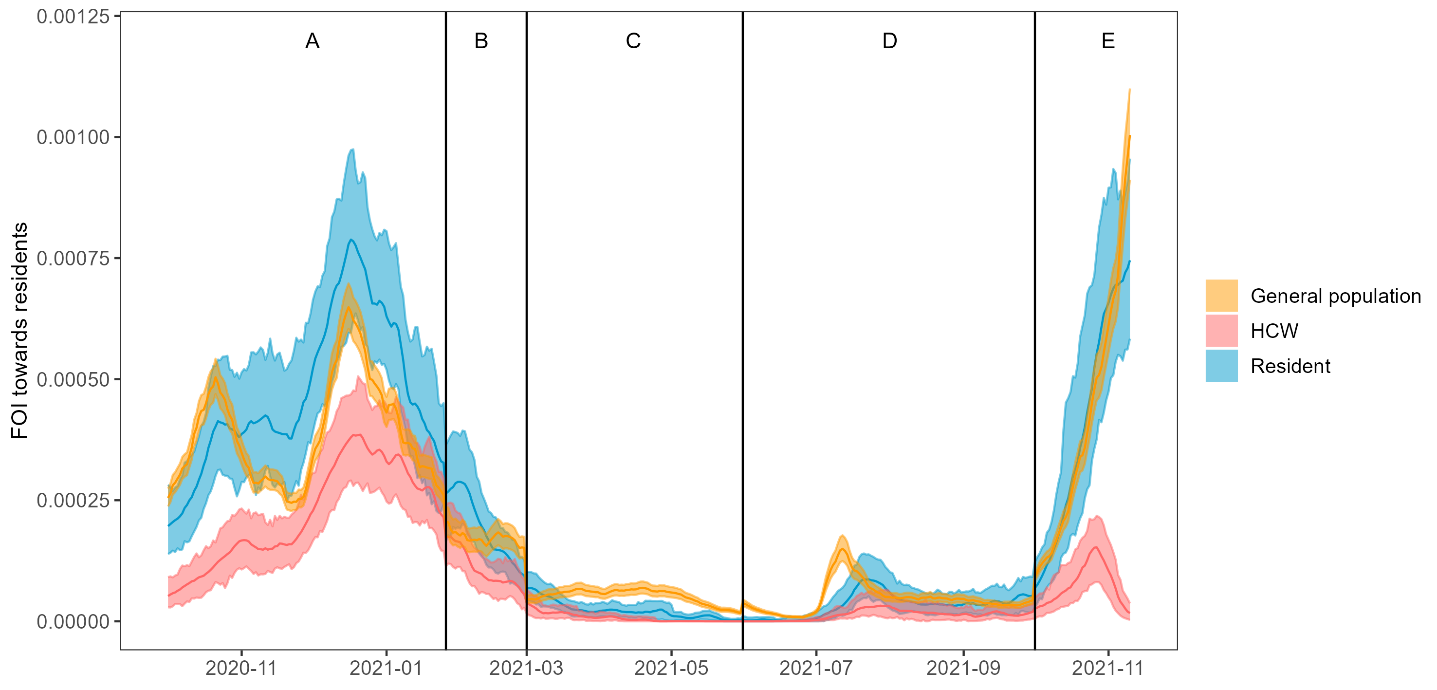


Supplementary Figure 18: Daily force of infection from each LTCF group towards residents for the alternative model assuming unequal resident contact fractions (30% residents, 60% HCW, 10% general populations). Dark lines indicate mean values. Shaded areas show 95% prediction intervals obtained by generating 1000 predictions by randomly sampling from the posterior distributions and datasets.

## References

1. McAloon C, Collins Á, Hunt K, et al (2020) Incubation period of COVID-19: a rapid systematic review and meta-analysis of observational research. BMJ Open 10:e039652

2. Hellewell J, Russell TW, Matthews R, et al (2021) Estimating the effectiveness of routine asymptomatic PCR testing at different frequencies for the detection of SARS-CoV-2 infections. BMC Med 19:1–10

3. Zhao S, Tang B, Musa SS, et al (2021) Estimating the generation interval and inferring the latent period of COVID-19 from the contact tracing data. Epidemics 36:100482

4. Leung KY, Metting E, Ebbers W, Veldhuijzen I, Andeweg SP, Luijben G, de Bruin M, Wallinga J, Klinkenberg D (2024) Effectiveness of a COVID-19 contact tracing app in a simulation model with indirect and informal contact tracing. Epidemics. https://doi.org/10.1016/J.EPIDEM.2023.100735

5. Besmettelijke mensen | Coronadashboard | Rijksoverheid.nl. https://coronadashboard.rijksoverheid.nl/landelijk/besmettelijke-mensen. Accessed 10 Aug 2023

6. Positief geteste mensen | Coronadashboard | Rijksoverheid.nl. https://coronadashboard.rijksoverheid.nl/landelijk/positieve-testen. Accessed 10 Aug 2023

7. Opnieuw meer medewerkers in verpleeghuizen | ActiZ. https://www.actiz.nl/opnieuw-meer-medewerkers-verpleeghuizen. Accessed 9 Aug 2023

8. Backer JA, Kassteele J Van de, Fakiri F El, Hens N, Wallinga J (2023) Contact patterns of older adults with and without frailties in the Netherlands during the COVID-19 pandemic. medRxiv 2023.05.09.23289550
